# Research and Analysis on the Localization of a 3-D Single Source in Lossy Medium Using Uniform Circular Array

**DOI:** 10.3390/s17061274

**Published:** 2017-06-02

**Authors:** Bing Xue, Xiaodong Qu, Guangyou Fang, Yicai Ji

**Affiliations:** 1 Key Laboratory of Electromagnetic Radiation and Sensing Technology, Institute of Electronics, Chinese Academy of Sciences, Beijing 100190, China; xuebing14@mails.ucas.ac.cn (B.X.); gyfang@mail.ie.ac.cn (G.F.); ycji@mail.ie.ac.cn (Y.J.); 2 University of Chinese Academy of Sciences, Beijing 100039, China

**Keywords:** uniform circular array, source localization, least square algorithm, lossy medium

## Abstract

In this paper, the methods and analysis for estimating the location of a three-dimensional (3-D) single source buried in lossy medium are presented with uniform circular array (UCA). The mathematical model of the signal in the lossy medium is proposed. Using information in the covariance matrix obtained by the sensors’ outputs, equations of the source location (azimuth angle, elevation angle, and range) are obtained. Then, the phase and amplitude of the covariance matrix function are used to process the source localization in the lossy medium. By analyzing the characteristics of the proposed methods and the multiple signal classification (MUSIC) method, the computational complexity and the valid scope of these methods are given. From the results, whether the loss is known or not, we can choose the best method for processing the issues (localization in lossless medium or lossy medium).

## 1. Introduction

Source localization has been attracting great attention for a long time, used widely in wireless communication, sonar and radar [1,2,3]. The direction-of-arrivals (DOAs) and location estimation have been solved by many researchers using algorithms such as MUSIC [4,5,6] and ESPRIT [7,8]. Although these algorithms yielded super accuracy for localization, calculation complexity is too great for single source localization [9]. For the single source location issue, in [10], the authors provided a simple and accurate algorithm to estimate two-dimensional angle with uniform circular array (UCA), although the algorithm is limited to even number of sensors. To overcome this restriction, the authors of [11] presented a generalized algorithm where the sensor number could be even or odd. The authors of [9] extended the work for a 3-D source location of a single source in [11], in which both the 2-D DOA and the range were estimated. The algorithm is more computationally efficient, and the performance is comparable with conventional 3-D MUSIC algorithm. Unfortunately, most previous works focused mainly on the problem of source localization in free space using an array of sensors. Recently, low frequency (<30 kHz) electromagnetic fields radiating from an underwater target were used as important signatures in target detection [12], where the source can be in conductive medium. In lossy medium, method such as the MUSIC algorithm may not work. Thus, new methods are necessary to process similar problems. Furthermore, UCA offers more advantages than other kinds of sensor arrays (two parallel uniform linear arrays (ULAs) [13] and an L-shaped ULA [14]), such as 360° azimuthal coverage, an identical directional pattern, and more angle information [15,16]. In this paper, a novel method is proposed to estimate the 3-D location information of a single source in lossy medium using UCA. The attenuation in the lossy medium has been taken into consideration. The novel solution includes four stages: (1) The target detection in lossy medium is considered in an array using scalar wave signal. We developed a mathematical model for this issue. (2) The phase information is applied to process the issue; then, the amplitude information is used to obtain location estimation, and the synthesis method using both the phase and amplitude information is present to process the issue in conductive medium. (3) Some situations, such as lossless medium (air), weak lossy medium (ionosphere), and conductive medium (ocean), are employed to test the performance of the proposed methods. (4) Whether the propagation loss is known or not, the valid scope of the proposed method and the MUSIC method are analyzed.

## 2. Mathematical Model

In lossy medium whose permeability, permittivity, and conductivity are μ,ε,σ, respectively, the propagation constant *k* of electromagnetic wave with frenqucy *f* can be writtern as follows:

(1) k=β+jα

where $\alpha =2\pi f\sqrt{\frac{\mu \varepsilon }{2}\left(\sqrt{1+{\left(\frac{\sigma }{2\pi f\varepsilon }\right)}^{2}}-1\right)}$, $\beta =2\pi f\sqrt{\frac{\mu \varepsilon }{2}\left(\sqrt{1+{\left(\frac{\sigma }{2\pi f\varepsilon }\right)}^{2}}+1\right)}$.

**Figure 1:**
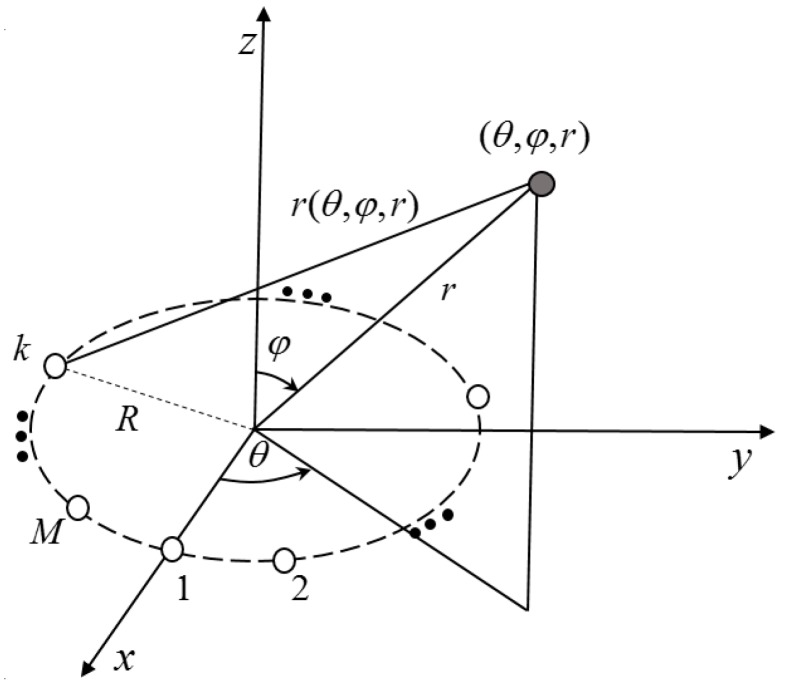
Single-source localization model using uniform circular array (UCA).

The UCA that contains *M* identical receiver sensors located on a circle array of radius *R* is impinged by electromagnetic field generated by a single narrow-band source. Both the UCA and the source are placed in the lossy medium. In a spherical coordinate system, the center of the UCA is employed as a reference point, located at the original point. All sensors are located on an *xoy* plane. The source is located at (r,θ,φ), where *r* is the distance between the reference point and the source, φ∈[0,π/2] is the elevation angle and θ∈[−π,π] is the azimuth angle. The model is shown in Figure 1.

Thus, the signal received by the *k*th sensor can be written as follows:

(2) $$x_{k}\left(t\right)=s\left(t\right)e^{j\left(\beta +j\alpha \right)\left(r-r_{k}\left(\theta ,\varphi ,r\right)\right)}+n_{k}\left(t\right)$$

where k=1,⋯,M is the *k*th number of the sensor. In the signal model, s(t) is the time sequence of the source signal, and n(t) denotes the time sequence of additional noise. In general, n(t) is independent of s(t) spatially and temporally. β+jα represents the propagation constant in lossy medium. $\Delta r_{k}=r-r_{k}\left(r,\theta ,\varphi \right)$ is the range difference between the distance from the source to the center of the UCA and the distance from the source to the *k*th sensor, which is given by

(3) $$r_{k}\left(\theta ,\varphi ,r\right)=\sqrt{r^{2}+R^{2}-2rR\cos\left(\gamma_{k}-\theta \right)\sin\varphi }$$

where $\gamma_{k}=2\pi \left(k-1\right)/M$ is the azimuth angle of the *k*th sensor.

Under the condition R<<r, $\Delta r_{k}$ can be approximated using Taylor series expansion. Thus, the signal model (Δt is suppressed for convenience) can be extended as

(4) $$x_{k}\left(t\right)=s\left(t\right)e^{j\left(\beta +j\alpha \right)\left(R\cos\left(\gamma_{k}-\theta \right)\sin\varphi -\frac{R^{2}}{2r}\left(1-\cos^{2}\left(\gamma_{k}-\theta \right)\sin^{2}\varphi \right)\right)}+n_{k}\left(t\right).$$

In matrix form, (4) can be written as

(5) X(t)=As(t)+N(t)

where **X** and **N** are M×1 dimensional signal and noise matrix. **A** is the steering vector.

(6) $$A(\theta ,\varphi ,r)=\left[\begin{array}{c} e^{j\left(\beta +j\alpha \right)\left(R\cos\left(\psi_{1}-\theta \right)\sin\varphi -\frac{R^{2}}{2r}\left(1-\cos^{2}\left(\psi_{1}-\theta \right)\sin^{2}\varphi \right)\right)}\\ e^{j\left(\beta +j\alpha \right)\left(R\cos\left(\psi_{2}-\theta \right)\sin\varphi -\frac{R^{2}}{2r}\left(1-\cos^{2}\left(\psi_{2}-\theta \right)\sin^{2}\varphi \right)\right)}\\ \vdots \\ e^{j\left(\beta +j\alpha \right)\left(R\cos\left(\psi_{M}-\theta \right)\sin\varphi -\frac{R^{2}}{2r}\left(1-\cos^{2}\left(\psi_{M}-\theta \right)\sin^{2}\varphi \right)\right)} \end{array}\right].$$

**S** is 1×K dimensional signal sequence at reference point with signal power of $\sigma_{s}^{2}$.

## 3. Proposed Method

Using Equation (5), the covariance matrix of **X** can be calculated:

(7) $$R=E\left\{X\cdot X^{H}\right\}=\sigma_{s}^{2}AA^{H}+\sigma_{n}^{2}I=\sigma_{s}^{2}D+\sigma_{n}^{2}I.$$

The superscript *H* is the conjugate transpose operator and $\sigma_{n}$ defines the sensor noise power.

(8) $$D=\exp\left\{-\alpha \left[\begin{array}{cccc} \Delta r_{1}+\Delta r_{1} & \Delta r_{1}+\Delta r_{2} & \cdots & \Delta r_{1}+\Delta r_{M}\\ \Delta r_{2}+\Delta r_{1} & \Delta r_{2}+\Delta r_{2} & \cdots & \Delta r_{2}+\Delta r_{M}\\ \vdots & \vdots & \vdots & \vdots \\ \Delta r_{M}+\Delta r_{1} & \Delta r_{M}+\Delta r_{2} & \cdots & \Delta r_{M}+\Delta r_{M} \end{array}\right]+j\beta \left[\begin{array}{cccc} \Delta r_{1}-\Delta r_{1} & \Delta r_{1}-\Delta r_{2} & \cdots & \Delta r_{1}-\Delta r_{M}\\ \Delta r_{2}-\Delta r_{1} & \Delta r_{2}-\Delta r_{2} & \cdots & \Delta r_{2}-\Delta r_{M}\\ \vdots & \vdots & \vdots & \vdots \\ \Delta r_{M}-\Delta r_{1} & \Delta r_{M}-\Delta r_{2} & \cdots & \Delta r_{M}-\Delta r_{M} \end{array}\right]\right\}.$$

The information of the source location (2-D DOA and range) appears in both the amplitude and phase of the sensors’ output.

### 3.1. Phase Method

We can use the phase information of **R** to estimate the source location. We can thus obtain

(9) $$\begin{array}{l} p_{mn}=\arg\left(R_{mn}\right)=\arg\left(D_{mn}\right)=\beta \left(\Delta r_{m}-\Delta r_{n}\right)\\ =\beta R{\left[\begin{array}{c} \cos\left(\gamma_{m}\right)-\cos\left(\gamma_{n}\right)\\ \sin\left(\gamma_{m}\right)-\sin\left(\gamma_{n}\right)\\ \cos\left(2\gamma_{m}\right)-\cos\left(2\gamma_{n}\right)\\ \sin\left(2\gamma_{m}\right)-\sin\left(2\gamma_{n}\right) \end{array}\right]}^{T}\times \left[\begin{array}{c} \cos\left(\theta \right)\sin\left(\varphi \right)\\ \sin\left(\theta \right)\sin\left(\varphi \right)\\ \frac{R}{4r}\cos\left(2\theta \right)\sin^{2}\left(\varphi \right)\\ \frac{R}{4r}\sin\left(2\theta \right)\sin^{2}\left(\varphi \right) \end{array}\right] \end{array}$$

where superscript *T* denotes the transpose operator. Assuming l=m−n, $p_{mn}$ is denoted in matrix form:

(10) p=Ub

where p, U, and b can be written as

(11) $$p={\left[p_{1,1+l},p_{2,2+l},p_{3,3+2l},\cdots ,p_{M-l,M}\right]}^{T}$$

(12) $$U={\left[\begin{array}{cccc} \cos\left(\gamma_{1}\right)-\cos\left(\gamma_{1+l}\right) & \cos\left(\gamma_{2}\right)-\cos\left(\gamma_{2+l}\right) & \cdots & \cos\left(\gamma_{M-l}\right)-\cos\left(\gamma_{M}\right)\\ \sin\left(\gamma_{1}\right)-\sin\left(\gamma_{1+l}\right) & \sin\left(\gamma_{2}\right)-\sin\left(\gamma_{2+l}\right) & \cdots & \sin\left(\gamma_{M-l}\right)-\sin\left(\gamma_{M}\right)\\ \cos\left(2\gamma_{1}\right)-\cos\left(2\gamma_{1+l}\right) & \cos\left(2\gamma_{2}\right)-\cos\left(2\gamma_{2+l}\right) & \cdots & \cos\left(2\gamma_{M-l}\right)-\cos\left(2\gamma_{M}\right)\\ \sin\left(2\gamma_{1}\right)-\sin\left(2\gamma_{1+l}\right) & \sin\left(2\gamma_{2}\right)-\sin\left(2\gamma_{2+l}\right) & \cdots & \sin\left(2\gamma_{M-l}\right)-\sin\left(2\gamma_{M}\right) \end{array}\right]}^{T}$$

(13) $$b=\beta R\left[\begin{array}{c} \cos\left(\theta \right)\sin\left(\varphi \right)\\ \sin\left(\theta \right)\sin\left(\varphi \right)\\ \frac{R}{4r}\cos\left(2\theta \right)\sin^{2}\left(\varphi \right)\\ \frac{R}{4r}\sin\left(2\theta \right)\sin^{2}\left(\varphi \right) \end{array}\right].$$

Then, ${\hat{R}}_{mn}=\left(1/N\right)\sum_{i=1}^{N}x_{m}\left(i\right)x_{n}\left(i\right)$ can estimate the $R_{mn}$, *N* is the sampling number, and the location of the source can be estimated using the least square algorithm.

(14) $$p={\left[p_{1,1+l},p_{2,2+l},p_{3,3+2l},\cdots ,p_{M-l,M}\right]}^{T}.$$

Therefore, the parameters of the source location are estimated as

(15) $$\begin{array}{l} \hat{\theta }=\arg\left({\hat{b}}_{1}+j{\hat{b}}_{2}\right)\\ \hat{\varphi }=\arcsin\left(\frac{1}{\beta R}\sqrt{{{\hat{b}}_{1}}^{2}+{{\hat{b}}_{2}}^{2}}\right)\\ \hat{r}=\frac{1}{4\beta }\frac{{{\hat{b}}_{1}}^{2}+{{\hat{b}}_{2}}^{2}}{\sqrt{{{\hat{b}}_{3}}^{2}+{{\hat{b}}_{4}}^{2}}} \end{array}.$$

### 3.2. Amplitude Method

Similarly, using the amplitude information of **R**,

(16) $$q_{mn}=\ln\left(\mathrm{abs}\left(R_{mn}\right)\right)=\ln\left(\sigma_{s}^{2}\right)+\alpha \left(\Delta r_{m}+\Delta r_{n}\right)\;\;q_{st}=\ln\left(\mathrm{abs}\left(R_{st}\right)\right)=\ln\left(\sigma_{s}^{2}\right)+\alpha \left(\Delta r_{s}+\Delta r_{t}\right)$$

where ln(⋅) represents the natural logarithm operator, and abs(⋅) denotes the absolute operator. Assuming n=s, then

(17) $$q_{mt}=q_{mn}-q_{nt}=\alpha R{\left[\begin{array}{c} \cos\left(\gamma_{m}\right)-\cos\left(\gamma_{t}\right)\\ \sin\left(\gamma_{m}\right)-\sin\left(\gamma_{t}\right)\\ \cos\left(2\gamma_{m}\right)-\cos\left(2\gamma_{t}\right)\\ \sin\left(2\gamma_{m}\right)-\sin\left(2\gamma_{t}\right) \end{array}\right]}^{T}\times \left[\begin{array}{c} \cos\left(\theta \right)\sin\left(\varphi \right)\\ \sin\left(\theta \right)\sin\left(\varphi \right)\\ \frac{R}{4r}\cos\left(2\theta \right)\sin^{2}\left(\varphi \right)\\ \frac{R}{4r}\sin\left(2\theta \right)\sin^{2}\left(\varphi \right) \end{array}\right].$$

If m−n=l,n−t=l, then m−t=2l. In matrix form,

(18) q=Vb

where

(19) $$q={\left[q_{1,1+2l},q_{2,2+2l},q_{3,3+2l},\cdots ,q_{M-2l,M}\right]}^{T}$$

(20) $$V={\left[\begin{array}{cccc} \cos\left(\gamma_{1}\right)-\cos\left(\gamma_{1+2l}\right) & \cos\left(\gamma_{2}\right)-\cos\left(\gamma_{2+2l}\right) & \cdots & \cos\left(\gamma_{M-2l}\right)-\cos\left(\gamma_{M}\right)\\ \sin\left(\gamma_{1}\right)-\sin\left(\gamma_{1+2l}\right) & \sin\left(\gamma_{2}\right)-\sin\left(\gamma_{2+2l}\right) & \cdots & \sin\left(\gamma_{M-2l}\right)-\sin\left(\gamma_{M}\right)\\ \cos\left(2\gamma_{1}\right)-\cos\left(2\gamma_{1+2l}\right) & \cos\left(2\gamma_{2}\right)-\cos\left(2\gamma_{2+2l}\right) & \cdots & \cos\left(2\gamma_{M-2l}\right)-\cos\left(2\gamma_{M}\right)\\ \sin\left(2\gamma_{1}\right)-\sin\left(2\gamma_{1+2l}\right) & \sin\left(2\gamma_{2}\right)-\sin\left(2\gamma_{2+2l}\right) & \cdots & \sin\left(2\gamma_{M-2l}\right)-\sin\left(2\gamma_{M}\right) \end{array}\right]}^{T}$$

(21) $$b=\alpha R\left[\begin{array}{c} \cos\left(\theta \right)\sin\left(\varphi \right)\\ \sin\left(\theta \right)\sin\left(\varphi \right)\\ \frac{R}{4r}\cos\left(2\theta \right)\sin^{2}\left(\varphi \right)\\ \frac{R}{4r}\sin\left(2\theta \right)\sin^{2}\left(\varphi \right) \end{array}\right].$$

Finally, the least square algorithm can be used to find the optimal solution.

(22) $$\hat{b}={\left[{\hat{b}}_{1}\;{\hat{b}}_{2}\;{\hat{b}}_{3}\;{\hat{b}}_{4}\right]}^{T}={\left(V^{T}V\right)}^{-1}V^{T}\hat{q}.$$

Using (22), the 2-D DOA and range of the source can be also estimated as follows:

(23) $$\begin{array}{l} \hat{\theta }=\arg\left({\hat{b}}_{1}+j{\hat{b}}_{2}\right)\\ \hat{\varphi }=\arcsin\left(\frac{1}{\alpha R}\sqrt{{{\hat{b}}_{1}}^{2}+{{\hat{b}}_{2}}^{2}}\right)\\ \hat{r}=\frac{1}{4\alpha }\frac{{{\hat{b}}_{1}}^{2}+{{\hat{b}}_{2}}^{2}}{\sqrt{{{\hat{b}}_{3}}^{2}+{{\hat{b}}_{4}}^{2}}} \end{array}.$$

### 3.3. Synthesis Method

Surely, in order to use the information in **R** adequately, we put the phase and amplitude information together to detect the source location in the conductive medium ($\alpha =\beta =\sqrt{\pi f\mu \sigma }$).

(24) $$\left[\begin{array}{c} p\\ q \end{array}\right]=\left[\begin{array}{c} U\\ V \end{array}\right]b.$$

Therefore, the least square solution can be obtained.

(25) $$\hat{b}={\left[{\hat{b}}_{1}\;{\hat{b}}_{2}\;{\hat{b}}_{3}\;{\hat{b}}_{4}\right]}^{T}={\left({\left[\begin{array}{c} U\\ V \end{array}\right]}^{T}\left[\begin{array}{c} U\\ V \end{array}\right]\right)}^{-1}{\left[\begin{array}{c} U\\ V \end{array}\right]}^{T}\left[\begin{array}{c} \hat{p}\\ \hat{q} \end{array}\right].$$

### 3.4. Applicability Analysis

When the propagation constants are known, the proposed methods can be applied in any situation for single-source location. In many situations, the character of the wave propagation is not accurately known. In the lossless medium, the MUSIC method can be used to estimate the source location, while this method is inefficient when the source is located in the lossy medium. The phase method can be used in any media. The performance of the phase method is close to that of the MUSIC method, dealing with the issue in the lossless medium. However, the results become worse along with the loss that increases in the lossy medium. The amplitude method uses the attenuated information to detect the source location, which gives a high accuracy in conductive medium. On the contrary, when the conductivity of the medium approaches zero (σ→0), the amplitude method gradually loses accuracy. Surely, the synthesis method applying more information than the other methods has high stability, when the source is located in the conductive medium.

We use the multiplication times to estimate the computational complexity. Assume *N* is the sampling number. The phase method needs O((M−l)N) multiplications to compute the covariance function and O(M) complexity to compute the least square algorithm. The amplitude method needs O(2(M−l)N) multiplications to compute the covariance function and O(M) complexity to compute the least square algorithm. The synthesis method needs O(3(M−l)N) multiplications to compute the covariance function and O(M) complexity to compute the least square algorithm. In addition, the MUSIC method has the highest computational complexity because of the space search.

## 4. Numerical Results

Five experiments are here conducted to show the performance of the proposed algorithm. In the simulation example, the UCA contains 12 sensors and l=1. To obtain stable results, 500 Monte Carlo tests runs are conducted. The simulation results are shown in root mean square errors (RMSEs). The signal-to-noise ratio (SNR) is defined relative to the signal.

**Figure 2:**
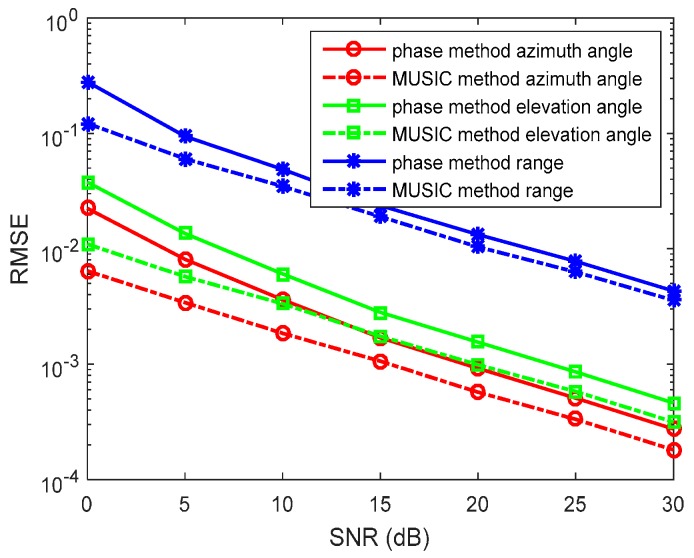
Root mean square errors (RMSEs) of location estimations for single-source in the lossless medium versus SNRs. $\left(r,\theta ,\varphi \right)=\left(1200,\frac{\pi }{6},\frac{\pi }{3}\right)$, and the snapshot number is 1000 with 500 independent trials.

**Figure 3:**
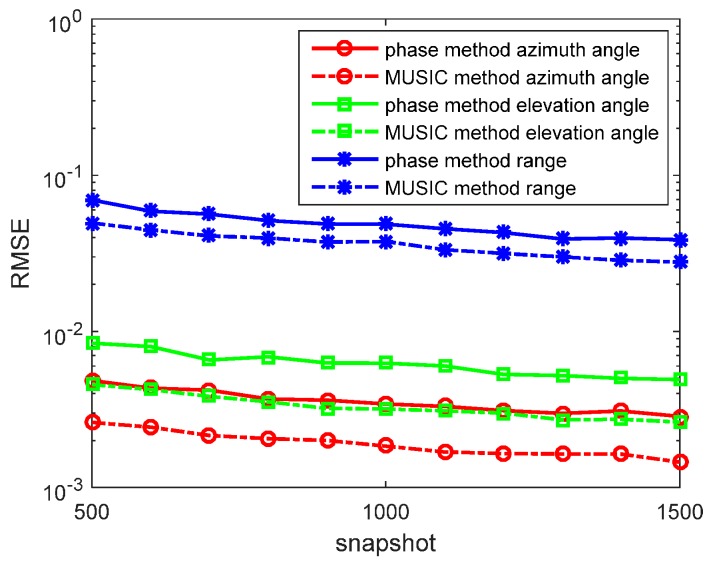
RMSEs of location estimations for single-source in the lossless medium versus snapshot. $\left(r,\theta ,\varphi \right)=\left(1200,\frac{\pi }{6},\frac{\pi }{3}\right)$, and the signal-to-noise ratio (SNR) is 10 dB with 500 independent trials.

In the first experiment, we set R=300 and $f=5\times 10^{5}\;\mathrm{Hz}$. The source is located at $\left(r,\theta ,\varphi \right)=\left(1200,\frac{\pi }{6},\frac{\pi }{3}\right)$ in the lossless medium system. We determine the SNR from 0 to 30 dB, containing *N* = 1000 snapshots in each run. The RMSEs of the azimuth angles, elevation angles, and the range estimations by the phase method are presented in Figure 2. We also determine the *N* from 500 to 1500 snapshots, containing SNR = 10 dB in each run. RMSEs of the azimuth angles, the elevation angles, and the range estimations by the phase method are presented in Figure 3. The amplitude method and the synthesis method are invalid in this situation. The MUSIC method has been studied in great detail as the reference.

In the figures, we can see that the accuracy of the phase method is similar to the MUSIC method in the lossless medium situation. For the azimuth angle, elevation angle, and range, the phase method can yield accurate results. At a high SNR, the accuracy of the phase method is closer to that of the MUSIC method. With snapshots increasing, the accuracy of the phase method becomes greater.

**Figure 4:**
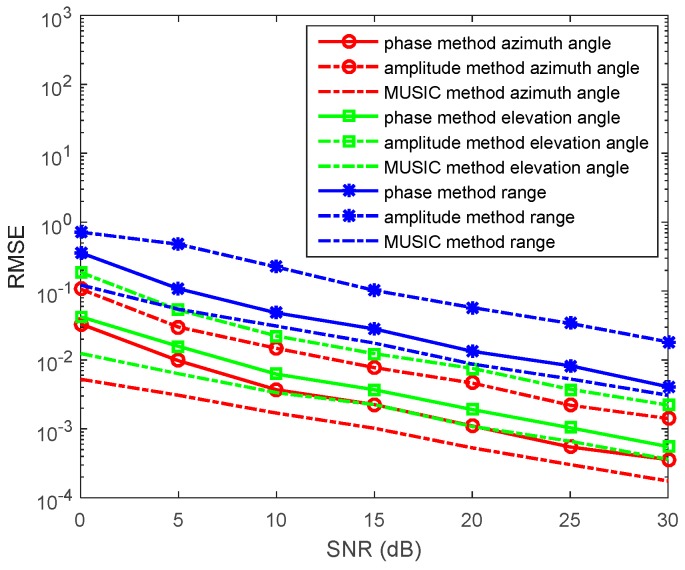
RMSEs of location estimations for single-source in the weak lossy medium versus SNRs. $\left(r,\theta ,\varphi \right)=\left(1200,\frac{\pi }{6},\frac{\pi }{3}\right)$, and the snapshot number is 1000 with 500 independent trials.

**Figure 5:**
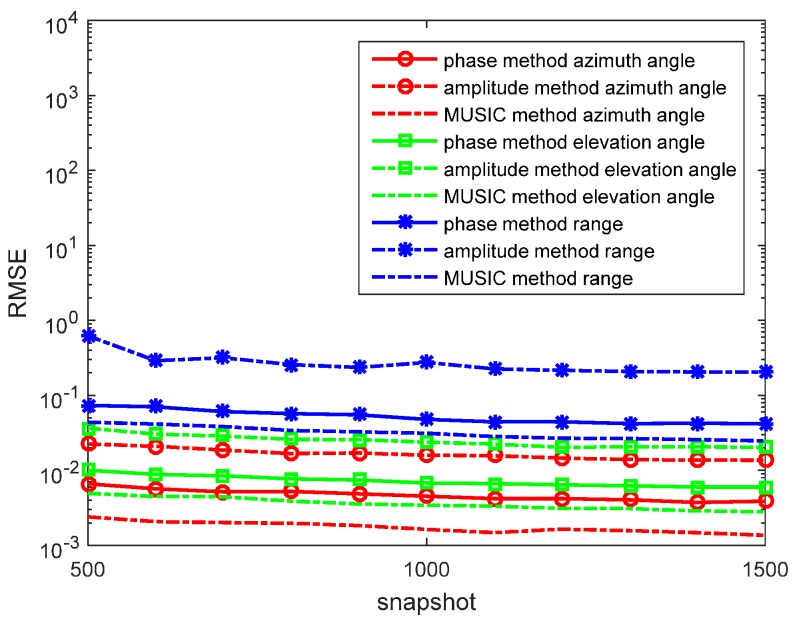
RMSEs of location estimations for single-source in the weak lossy medium versus snapshot. $\left(r,\theta ,\varphi \right)=\left(1200,\frac{\pi }{6},\frac{\pi }{3}\right)$, and the SNR is 10 dB with 500 independent trials.

In the second experiment, we set R=300, $f=5\times 10^{5}\;\mathrm{Hz}$, and $\sigma =10^{-5}$. When $\varepsilon =\varepsilon_{0}=8.8541878\times 10^{-12}F/m$, $2\pi f\varepsilon \approx 2.78\times 10^{-5}>\sigma$. The source is located at $\left(r,\theta ,\varphi \right)=\left(1200,\frac{\pi }{6},\frac{\pi }{3}\right)$ in a weak lossy medium system (such as ionosphere). We determine the SNR from 0 to 30 dB, containing *N* = 1000 snapshots. The RMSEs of the azimuth angles, elevation angles, and the range estimations by the phase method and amplitude method are presented in Figure 4. We also determine *N* from 500 to 1500 snapshots, containing SNR = 10 dB in each run. RMSEs of the azimuth angles, elevation angles, and the range estimations by the phase method and amplitude method are presented in Figure 5. The MUSIC method has been studied in great detailas the reference. The synthesis method is invalid because α≠β.

In the figures, we can see that the accuracy of the phase method is higher than that of the amplitude method in the weak lossy medium. With snapshots increasing, the accuracy of the phase method and amplitude method become greater. However, the MUSIC method still has an accuracy that is greater than that of the phase method and amplitude method.

**Figure 6:**
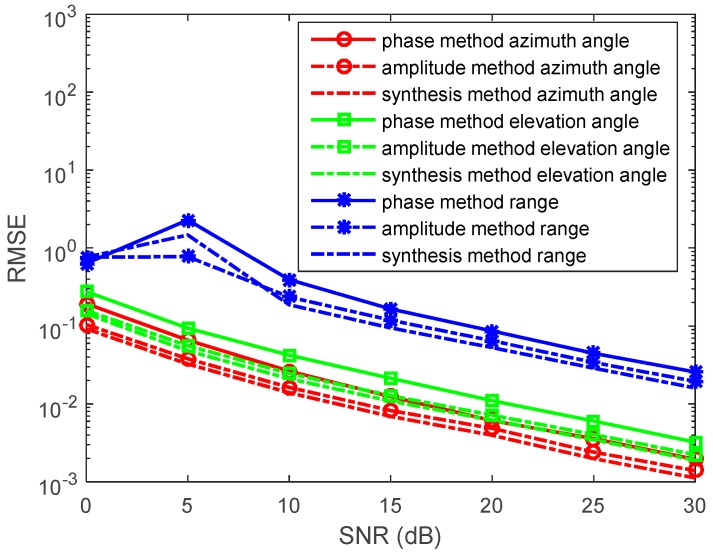
RMSEs of location estimations for single-source in the conductive medium versus SNRs. $\left(r,\theta ,\varphi \right)=\left(120,\frac{\pi }{6},\frac{\pi }{3}\right)$, and the snapshot number is 1000 with 500 independent trials.

**Figure 7:**
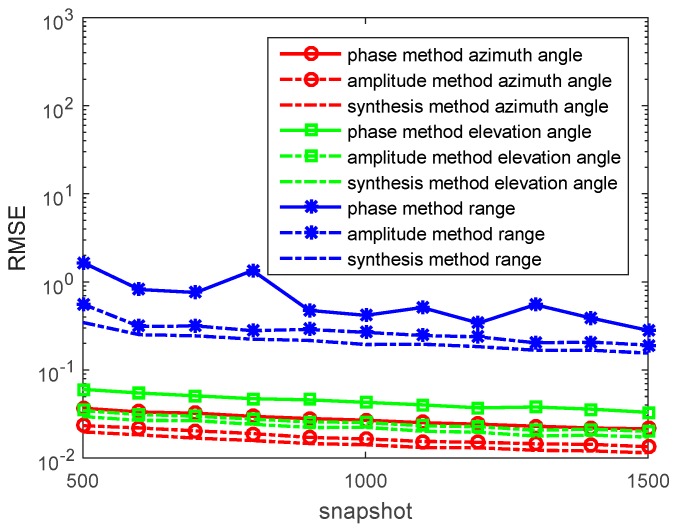
RMSEs of location estimations for single-source in the conductive medium versus snapshot. $\left(r,\theta ,\varphi \right)=\left(120,\frac{\pi }{6},\frac{\pi }{3}\right)$, and the SNR is 10 dB with 500 independent trials.

In the third experiment, we set R=30, f=20Hz, and σ=4. When $\varepsilon =80\varepsilon_{0}$, $2\pi f\varepsilon \approx 2.22\times 10^{-3}<<\sigma$ and $\alpha =\beta =\sqrt{\pi f\mu \sigma }$. The source is located at $\left(r,\theta ,\varphi \right)=\left(120,\frac{\pi }{6},\frac{\pi }{3}\right)$ in a conductive medium system (such as ocean). We determine the SNR from 0 to 30 dB, containing *N* = 1000 snapshots. The RMSEs of the azimuth angles, elevation angles, and the range estimations by the phase method, the amplitude method, and the synthesis method are presented in Figure 6. We also determine the *N* from 500 to 1500 snapshots, containing SNR = 10 dB in each run. RMSEs of the azimuth angles, elevation angles, and the range estimations by the phase method, the amplitude method, and the synthesis method are presented in Figure 7.

In the figures, we can see that the accuracy of the amplitude method is higher than that of the phase method in the conductive medium. The synthesis method has the highest accuracy. With snapshots increasing, the accuracy of the phase method, the amplitude method, and the synthesis method becomes greater.

**Figure 8:**
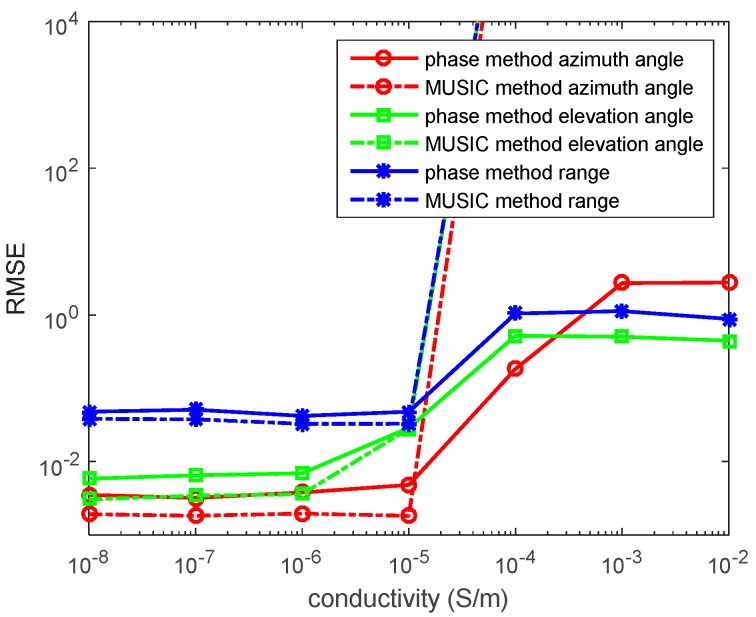
RMSEs of location estimations for single-source in the lossy medium versus snapshot. $\left(r,\theta ,\varphi \right)=\left(1200,\frac{\pi }{6},\frac{\pi }{3}\right)$, σ is unknown, the SNR is 10 dB, and the snapshot number is 1000 with 500 independent trials.

In the fourth experiment, we set R=300, $f=5\times 10^{5}\mathrm{Hz}$, *N* = 1000 snapshots and SNR = 10 dB. When $\varepsilon =\varepsilon_{0}$, $2\pi f\varepsilon \approx 2.78\times 10^{-5}$. The source is located at $\left(r,\theta ,\varphi \right)=\left(1200,\frac{\pi }{6},\frac{\pi }{3}\right)$ in the lossy medium system. We determine σ from $10^{-8}\;S/m$ to $10^{-2}\;S/m$. We assume σ is unknown (the location estimations are based on the lossy medium). The RMSEs of the azimuth angles, elevation angles, and the range estimations by the phase method and the MUSIC method are presented in Figure 8.

In the figure, for the phase method, the location estimations in the low conductive medium are relatively accurate, while the location estimations in the high conductive medium yield a low accuracy. The MUSIC method is invalid for estimating the source location in the high conductive medium.

**Figure 9:**
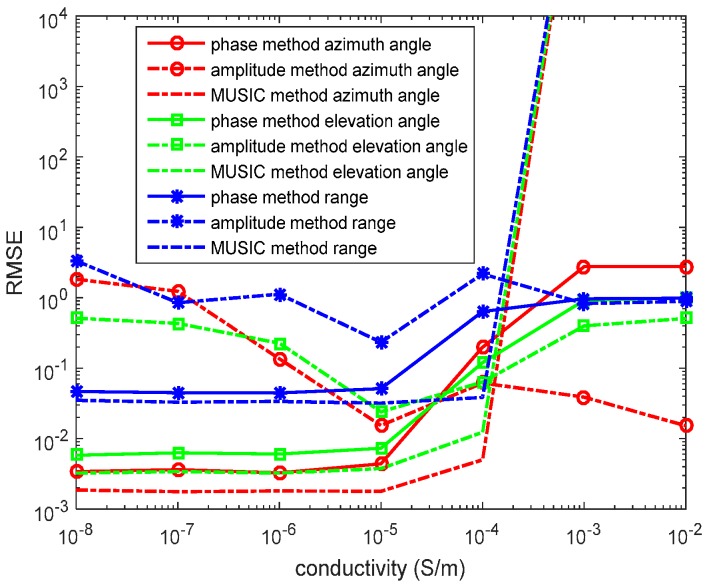
RMSEs of location estimations for single-source in the lossy medium versus snapshot. $\left(r,\theta ,\varphi \right)=\left(1200,\frac{\pi }{6},\frac{\pi }{3}\right)$, σ is known, the SNR is 10 dB, and the snapshot number is 1000 with 500 independent trials.

In the fifth experiment, we set R=300, $f=5\times 10^{5}\mathrm{Hz}$, *N* = 1000 snapshots and SNR = 10 dB. When $\varepsilon =\varepsilon_{0}$, $2\pi f\varepsilon \approx 2.78\times 10^{-5}$. The source is located at $\left(r,\theta ,\varphi \right)=\left(1200,\frac{\pi }{6},\frac{\pi }{3}\right)$ in the lossy medium system. We determine σ from $10^{-8}\;S/m$ to $10^{-2}\;S/m$. We assume σ is known. The RMSEs of the azimuth angles, elevation angles, and the range estimations by the phase method, the amplitude method, and the MUSIC method are presented in Figure 9.

In the figure, for the phase method, the location estimations in the low conductive medium are relatively accurate, while the location estimations in the high conductive medium yield a low accuracy. The MUSIC method is invalid for estimating the source location in the high conductive medium. However, the valid scope of the MUSIC method with a known σ is wider than that of the MUSIC method with an unknown σ, which we can infer from Figure 8 and Figure 9. The amplitude method has an accuracy lower than that of the phase method in low conductivity, but its results are the reverse in high conductivity.

## 5. Conclusions

This paper thus presents the model and methods for 3-D single source localization in lossy medium using UCA. It also analyzes the valid scope and computational complexity of the proposed methods and the MUSIC method. We also provide results of these methods in lossless medium (air), weak lossy medium (ionosphere), and conductive medium (ocean). In low conductivity (2πfε>σ), the MUSIC method is valid, and the phase method has an accuracy that is higher than that of the amplitude method. In high conductivity (2πfε<σ), the MUSIC method is invalid, and the phase method has an accuracy lower than that of the amplitude method. Of course, in the conductive medium (2πfε<<σ), the synthesis method has the highest accuracy compared to the phase method and the amplitude method.
